# Associations between proximity and density of local alcohol outlets and alcohol use among Scottish adolescents

**DOI:** 10.1016/j.healthplace.2012.10.004

**Published:** 2013-01

**Authors:** Robert Young, Laura Macdonald, Anne Ellaway

**Affiliations:** MRC/CSO Social and Public Health Sciences Unit, University of Glasgow, 4 Lilybank Gardens, Glasgow G12 8RZ, Scotland, UK

**Keywords:** GIS (geographic information systems), Alcohol, Adolescents, Alcohol outlets, Outlet density

## Abstract

Associations between different alcohol outcomes and outlet density measures vary between studies and may not be generalisable to adolescents. In a cross-sectional study of 979 15-year old Glaswegians, we investigated the association between alcohol outlet availability (outlet density and proximity), outlet type (on-premise vs. off-premise) and frequent (weekly) alcohol consumption. We adjusted for social background (gender, social class, family structure). Proximity and density of on-premise outlets were not associated with weekly drinking. However, adolescents living close (within 200 m) to an off-sales outlet were more likely to drink frequently (OR 1.97, *p*=0.004), as were adolescents living in areas with many nearby off-premises outlets (OR 1.60, *p*=0.016). Our findings suggest that certain alcohol behaviours (e.g. binge drinking) may be linked to the characteristics of alcohol outlets in the vicinity.

## Highlights

► We examined the link between adolescent alcohol use and alcohol outlet availability. ► Proximity and density of *on-premises* outlets is unrelated to adolescent alcohol use. ► Adolescents in close proximity to *off-licence* outlets have elevated alcohol use.

## 1 Introduction

Over the past two decades both alcohol consumption (WHO Regional Office for Europe, 2010) and alcohol-related problems (liver cirrhosis, binge-drinking, alcohol-related violence and alcohol-related deaths) among adults have increased (MacNaughton and Evelyn, 2011, Room et al., 2005, Room et al., 2011) along with growing concern about parallel rises in problematic drinking among adolescents. There is increasing public health concern about how excessive and problem drinking among adolescents may be exacerbated by exposure to environments which facilitate access to alcohol (Bryden et al., 2011). The European region is designated the ‘Heaviest drinking region in the world’ (WHO Regional Office for Europe, 2010) and when ranked by overall alcohol-risk score and frequency of binge drinking the UK is among the most problematic drinking nations within Western Europe (Anderson and Baumberg, 2006). Based on 2007 commercial sales, Scotland ranks as the eighth heaviest alcohol consumer in the world with the majority of sales purchased at off-premises (off-sales) outlets (McNeill, 2009). Similarly, within the European context British adolescents are among the most excessive alcohol users and problematic drinkers (Hibell et al., 2009). A recent Scottish government review of the links between off-sales outlets, excessive underage drinking and alcohol-related trouble acknowledges such relation ships are likely to be complex and non-direct, but concludes that researchers outside North America may have ‘missed’ the importance of alcohol outlets as a major influence on alcohol consumption (Pattoni et al., 2007).

Adolescents are an important age group to focus upon as drinking patterns are being established (potentially with long-term effects) and both adolescents and young adults are considered disproportionately heavy targets of alcohol advertising (Pattoni et al., 2007). One plausible explanation for this growth in consumption, especially among adolescents, is the increased opportunity to access alcohol locally.

A key factor that may facilitate alcohol consumption is availability or ‘ease of access’, with research focusing on the impact of outlet density (the number of alcohol outlets within a particular area) or type of outlet (on- or off-premise drinking; Hay et al., 2009). Reviewing the evidence The (American) Task Force on Community Preventive Services (2009) concluded ‘*There was sufficient evidence to recommend controlling the density and nature of alcohol outlets by regulatory authority* (e.g.*, licensing and zoning) as a means of reducing or controlling excessive alcohol consumption and related harms*’. They also note that (on balance) alcohol outlet density tends to be higher in socially deprived neighbourhoods. In contrast a Californian study by Pollack et al. (2005), while confirming that deprived areas had the greatest number of local alcohol outlets, found that those living in the least deprived areas had the *highest* levels of alcohol consumption. One recurring difficulty is that much of the research comes from North America and may not be generalisable to other contexts, although a contemporary study using aggregate local council-level data in England also links outlet density with alcohol-related problems (Coghill, 2011).

A recent systematic review of the influence of alcohol availability on alcohol use identified only five studies with outcomes for adolescents (Bryden et al., 2011). All five studies reported some association with alcohol use and outlet density, but the nature of the relationship varied considerably by individual study. Three studies looked at the relationship between off-premise outlet density and alcohol use, two found associations with both drinking and heavy drinking (Chen et al., 2010, Rootman and Oakey, 1973) and one found no association (Kuntsche et al., 2008). Two additional studies found alcohol use linked to overall outlet density; in one study (of on- and off-premises outlet density) this was only significant for heavy drinking (Truong and Sturm, 2009), in the other only with total quantity consumed (Huckle et al., 2008). Finally, one previously mentioned study found an association between density of on-premises outlets and drinking, but not heavy drinking (Kuntsche et al., 2008). Both studies conducted outside North America (New-Zealand (Huckle et al., 2008) Switzerland (Kuntsche et al., 2008)) report conflicting results.

The conflicting literature may be explained by differences in how ‘availability of alcohol’ is measured. Most studies count the number of outlets in a particular administrative district, some measure the number of outlets within a certain radius of participant's home address or regular travel route, a few measure the proximity of residence to nearest outlet—as the crow flies or via accessible road networks. Studies that actually measure adolescence exposure to passing outlets by tracking their daily route using global positioning satellite technology remain a minority (Basta et al., 2010). Importantly, research has concentrated on adults, yet given the qualitative difference in adult and adolescent drinking behaviour may not be entirely relevant. For example, in Scotland and in many other nations it is illegal for those under the age of 18 to purchase alcohol, thus adolescents typically use a different range of practices and outlets to access alcohol than adult drinkers (MacNaughton and Evelyn, 2011).

Throughout adolescence and adulthood, males tend to drink more than females and there is evidence that for both biological and social reasons each gender may behave differently when intoxicated (Young et al., 2008). Accordingly, it is important to investigate the interaction between gender and alcohol availability. Family structure and young people's patterns of alcohol use are interrelated (Foxcroft and Lowe, 1991); while at the same time household composition (proportion of one-parent households) is a component of some indexes of neighbourhood deprivation and fragmentation. A neighbourhood is sometimes characterised by the social class composition of its population and there is evidence that social class is associated with differing patterns of alcohol use (Norstrom and Romelsjo, 1998). Consequently, it is important to include both family structure and social class as potential confounders.

**Fig. 1:**
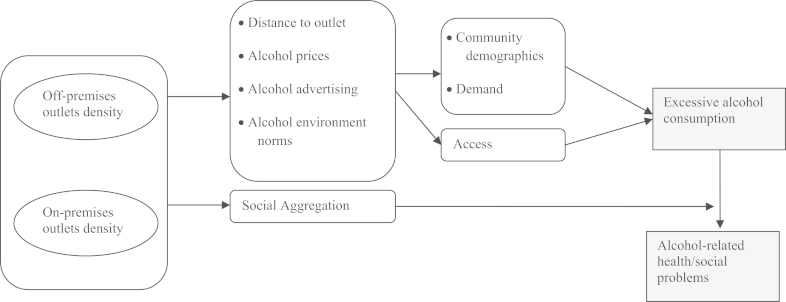
Simplified hypothetical model of the pathways between outlet density, excessive alcohol consumption and alcohol-related harm (adapted from Campbell et al. 2009).

In general, the alcohol outlet literature developed as an atheoretical response to a public health concern, rather than developing from any particular theoretical perspective. However, Campbell et al. (2009) outline an analytical model which draws together the key factors and sketches the major pathways linking modifiable alcohol outlet density factors to health outcomes (Fig. 1). We used this model to guide our study.

## 2 Aim

This paper aims to measure the association between alcohol consumption among adolescents (aged 15) and the availability of alcohol outlets, measured by both proximity, density, and type of outlet, while adjusting for social background (social class and family structure) and investigating gender interactions.

## 3 Methods

### 3.1 Sample

Data came from a subsample of 979 adolescents, drawn from a larger broadly representative school-based study of 3194 15-year olds from 22 schools conducted in 2006; full details of the design sample and ethical approval are provided elsewhere (Sweeting et al., 2008). The subsample comprised all pupils in the study who resided within the geographical boundary of Glasgow City Council. Only pupils resident within Glasgow were included in this study as reliable data on licensed premises was only available for outlets within Glasgow City. Pupils came from a wide range of social backgrounds and neighbourhoods within Glasgow. We excluded seven cases where less than five pupils attended a single school, pupils attending private/independent schools (66 pupils) and those with missing data (42 pupils), reducing the final sample to 868 cases from 11 schools.

### 3.2 Measures

#### 3.2.1 Data zones

Look-up tables were used to link each adolescents unit postcode address to Scottish data zones, the key small-area statistical geography in Scotland (Scottish Executive, 2004). Data zones are groups of 2001 Census output areas and the majority have populations between 500 and 1000 residents. They nest within local government boundaries, and where possible, they respect physical boundaries and natural communities, have a regular shape and contain households with similar social characteristics. There are 694 data zones in the Glasgow City Council boundary, with a mean population of 832 (range 248–2243) (Scottish Executive, 2004).

#### 3.2.2 Mapping alcohol outlets

A list of alcohol outlets in Glasgow City with street addresses was obtained from Glasgow City Council in 2006. The list included seven categories of outlet: public houses, off-sales (including super-markets), private members' clubs (e.g. social clubs, sports clubs, student unions, etc.), entertainment (e.g. bingo halls, casinos, concert halls, nightclubs, etc.), restaurants, refreshment (cafe style premises where alcohol may be served with food) and hotels. If an outlet had two types of license, e.g. public house and off-sales, they were included once in the analysis with all outlets together, but included both in the public house analysis and off-sales analysis. We chose to combine clubs, entertainment, restaurants, refreshments and hotels because of small numbers, details reported elsewhere (Ellaway et al., 2010).

#### 3.2.3 Alcohol outlet, type, density and proximity

We distinguished between four types of outlet (1) public houses (2) off-sales (3) ‘other’ alcohol outlets (clubs, entertainment, restaurants, refreshments and hotels combined) and (4) all outlets combined. Measures were calculated separately for each type of outlet. We calculated the total count of outlets in each data zone, coded as 0, 1, 2, or 3+ for each category of outlet.

Network analysis (i.e. finding the shortest path between two locations on a road network) was carried out for each outlet using Arc GIS version 9.1. Streetmaps (including point addresses) were obtained from UK Ordnance Survey (Ordnance Survey, 2006). Every outlet and participant address was geocoded by unit postcode. Network analysis was undertaken to calculate the distance (in metres) between each participant's postcode and their nearest outlet. This was coded: 0–200, 200.01–400, 400.01–600, 600.01–800, or 800.01+ metres from nearest outlet. Additionally for each type of outlet, we calculated the number within 1200 m distance of participants postcode, this represents an approximately 15 min walk. This was recoded into four approximately equal categories containing 0–10, 11–20, 21–30 or 31+ local off-sales outlets and 0–3, 4–9, 10–19 or 20+ local public houses (pubs).

#### 3.2.4 Weekly alcohol use and social background

Pupils self-reported alcohol use was assessed by asking ‘How often do you have an alcoholic drink (not just a sip)?’, measured on a seven-point frequency scale (‘every day’ to ‘I never had an alcoholic drink’). This was dichotomised into weekly drinking (drink at least once a week) vs. less frequent use (including non-drinkers). Self-reported social background measures included gender and family structure: coded as 2-parent, 1-parent, reconstituted (one ‘birth’ parent and new partner) or other (relative, foster parent, or other carer). Social class of the head of household, was derived from data about parental occupation obtained in a brief pupil interview. This was initially coded using the UK Registrar General's classification system, based on father's current occupation or, if absent or not working, mother's occupation (ONS, 2000 and recoded into manual, non-manual and missing categories.

#### 3.2.5 Statistical analysis

The analysis used logistic regression to determine the association between measures of density, proximity and weekly alcohol use, with separate analyses conducted for each of the outlet types. Analysis was conducted firstly unadjusted and then adjusted for social background, i.e. gender, family structure and social class. Further analyses included interaction terms between gender and outlet density or proximity. We conducted analysis both unadjusted and adjusted for the clustering attributable to the school sampling design using iterative generalized least squares estimation within the multilevel software package MLwiN 2.20 (Rasbash et al., 2009).

## 4 Results

Significant associations were found only for alcohol use and off-sales density and proximity and consequently we report full results for only off-sales outlets. Descriptive statistics (*n*, %) for all of the study variables are presented in Table 1, along with the proportion who drink weekly in each category. Just over half (57.9%) of the participants reported that they drank weekly or more frequently and a minority drank every day (16.1%). Network distance to nearest off-sales outlet, number of off-sale outlets within 1200 m, gender and family structure were all associated with weekly alcohol use. Association between accessibility measures and other outlet types were non-significant. To illustrate this we report the non-significant association between weekly alcohol use and network distance to the nearest public house, number of public houses within 1200 m, and number of public houses in participants' data zone. The relationship (odds ratio) between weekly drinking and network distance to nearest off-sales outlet is significant when unadjusted or adjusted for social background and remained significant after further adjustment for sample clustering. Pupils living close (within 200 m) to an off-sales outlet were nearly twice as likely to drink weekly, than those living more than 800 m away from such outlets (*p* ≤004, Table 2). There was no association between outlet density (as measured by the number of off-sales outlets in participants data zone) in the unadjusted or adjusted models (Table 3). Pupils living in areas with a high density of nearby off-sales outlets (31+ within 1200 m) were approximately 50% more likely to drink weekly than those with few (0–10) off-sales outlets within 1200 m (*p*≤05, Table 4), although this was less significant in the mutually adjusted (clustered) model (*p*≤0.095, Table 4). There were no significant interactions between gender, outlet proximity or density.

**Table 1:** Descriptive statistic.

	**Frequency**		**Drink <weekly**		**Drink weekly**		***P***
**Categorical variables**	***N***	**%**	***N***	**%**	***N***	**%**	

**Drink weekly**							
No	365	42.1	–	–	–	–	
Yes	503	57.9	–	–	–	–	
**Nearest off-sales (m)**							
800.01+	132	15.2	69	18.9	63	12.5	
600.01–800	111	12.8	47	12.9	64	12.7	
400.01–600	182	21.0	76	20.8	106	21.1	
200.01–400	261	30.1	108	29.6	153	30.4	
0–200	182	21.0	65	17.8	117	23.3	0.069; trend=0.007
**N of off-sales within 1200 m**							
0–10	206	23.7	100	27.4	106	21.1	
11–20	212	24.4	91	24.9	121	24.1	
21–30	226	26.0	91	24.9	135	26.8	
31+	224	25.8	83	22.7	141	28.0	0.101; trend=0.014
**N off-sales in data zone**							
0	515	59.3	224	61.4	291	57.9	
1	171	19.7	68	18.6	103	20.5	
2	114	13.1	46	12.6	68	13.5	
3+	68	7.8	27	7.4	41	8.2	0.778
**Nearest pub (metres)**							
800.01+	266	30.6	120	32.9	146	29.0	
600.01–800	121	13.9	55	15.1	66	13.1	
400.01–600	185	21.3	77	21.1	108	21.5	
200.01–400	184	21.2	67	18.4	117	23.3	
0–200	112	12.9	46	12.6	66	13.1	0.398
**N of pubs within 1200 m**							
0–3	178	20.5	80	21.9	98	19.5	
4–9	224	25.8	100	27.4	124	24.7	
10–19	227	26.2	94	25.8	133	26.4	
20+	239	27.5	91	24.9	148	29.4	0.421
**N pubs in data zone**							
0	623	71.8	262	71.8	361	71.8	
1	169	19.5	72	19.7	97	19.3	
2	41	4.7	20	5.5	21	4.2	
3+	35	4.0	11	3.0	24	4.8	0.493
**Gender**							
Male	432	49.8	164	44.9	268	53.3	
Female	436	50.2	201	55.1	235	46.7	0.015
**Social class**							
Missing	149	17.2	59	16.2	90	17.9	
Manual	363	41.8	168	46.0	195	38.8	
Non-manual	356	41.0	138	37.8	218	43.3	0.100
**Family structure**							
2-parent	555	63.9	202	55.3	353	70.2	
1-Parent	93	10.7	50	13.7	43	8.5	
Reconstituted/Other	220	25.3	113	31.0	107	21.3	<0.001

979 cases in Glasgow city council area; exclude schools under 5 pupils (*n*=972); exclude Private/independent school (66 cases); exclude missing data (42 missing cases); final sample=868.

**Table 2:** Relationship (odds ratio) between weekly drinking and distance to nearest off-sales outlet at age 15.

**Predictor**	**Standard estimates**				**Adjusted for sample clustering**			
	**Weekly drinking unadjusted OR**	***p***	**Weekly drinking adjusted OR**	***p***	**Weekly drinking unadjusted OR**	***p***	**Weekly drinking adjusted OR**	***p***

**Nearest off-sales (metres)**								
800.01+	1.00		1.00		1.00		1.00	
600.01–800	1.49 (0.90–2.48)	0.123	1.41 (0.84–2.38)	0.193	1.48 (0.88–2.46)	0.136	1.41 (0.83–2.38)	0.200
400.01–600	1.53 (0.97–2.40)	0.066	1.48 (0.93–2.34)	0.098	1.52 (0.97–2.40)	0.070	1.48 (0.93–2.36)	0.100
200.01–400	1.55 (1.02–2.36)	0.041	1.49 (0.97–2.30)	0.069	1.51 (0.99–2.32)	0.057	1.46 (0.94–2.26)	0.089
0–200	1.97 (1.25–3.11)	0.004	2.00 (1.25–3.20)	0.004	1.93 (1.21–3.08)	0.006	1.98 (1.23–3.19)	0.005
**Sociodemographic confounders**								
**Family (2-parent)**	1.00		1.00		1.00		1.00	
1-Parent	0.49 (0.32–0.77)	0.002	0.52 (0.33–0.82)	0.005	0.50 (0.32–0.79**)**	0.003	0.54 (0.34–0.85)	0.008
Reconstituted/Other	0.54 (0.40–0.74)	≤0.001	0.45 (0.31–0.64)	≤0.001	0.55 (0.40–0.76**)**	≤0.001	0.47 (0.33–0.66)	≤0.001
**Social Class (non-manual)**	1.00		1.00		1.00		1.00	
Missing	0.97 (0.65–1.43)	0.861	1.32 (0.86–2.03)	0.209	0.91 (0.61–1.36**)**	0.643	1.24 (0.80–1.92)	0.329
Manual	0.73 (0.55–0.99)	0.042	0.70 (0.52–0.96)	0.025	0.70 (0.52–0.95**)**	0.020	0.68 (0.50–0.92)	0.013
**Gender (male)**	1.40 (1.07–1.83)	0.015	1.32 (1.00–1.75)	0.049	1.42 (1.08–1.86**)**	0.011	1.34 (1.01–1.78)	0.040

**Table 3:** Relationship (odds ratio) between numbers of off-sales outlets in data zone at age 15.

**Predictor**	**Standard estimates**				**Adjusted for sample clustering**			
	**Weekly drinking unadjusted OR**	***p***	**Weekly drinking adjusted OR**	***p***	**Weekly drinking unadjusted OR**	***p***	**Weekly drinking adjusted OR**	***p***

**N of off-sales in data zone**								
0	1.00		1.00		1.00		1.00	
1	1.17 (0.82–1.66)	0.393	1.15 (0.80–1.64)	0.459	1.17 (0.82–1.67)	0.374	1.15 (0.80–1.65)	0.446
2	1.14 (0.75–1.72)	0.540	1.22 (0.80–1.87)	0.357	1.12 (0.73–1.70)	0.610	1.19 (0.77–1.82)	0.439
3+	1.17 (0.70-1.96)	0.553	1.26 (0.74–2.14)	0.402	1.10 (0.66–1.85)	0.706	1.20 (0.70–2.04)	0.508
**Sociodemographic confounders**								
**Family (2-parent)**	1.00		1.00		1.00		1.00	
1-Parent	0.49 (0.32–0.77)	0.002	0.51 (0.33–081)	0.004	0.50 (0.32–0.79)	0.003	0.53 (0.34–0.84)	0.007
Reconstituted/Other	0.54 (0.40–0.74)	≤0.001	0.45 (0.31–0.64)	≤0.001	0.55 (0.40–0.76)	≤0.001	0.47 (0.33–0.66)	≤0.001
**Social class (non-manual)**	1.00				1.00		1.00	
Manual	0.97 (0.65–1.43)	0.861	1.33 (0.86–2.04)	0.199	0.91 (0.61–1.36)	0.643	1.26 (0.81–1.94)	0.306
Missing	0.73 (0.55–0.99)	0.042	0.70 (0.52-0.95)	0.023	0.70 (0.52–0.95)	0.020	0.68 (0.50–0.92)	0.014
**Gender (male)**	1.40 (1.07–1.83)	0.015	1.31 (0.99–1.73)	0.056	1.42 (1.08–1.86)	0.011	1.33 (1.00–1.76)	0.047

**Table 4:** Relationship (odds ratio) between numbers of off-sales outlets within 1200 m of residence at age 15.

**Predictor**	**Standard estimates**				**Adjusted for sample clustering**			
	**Weekly drinking unadjusted OR**	***p***	**Weekly drinking adjusted OR**	***p***	**Weekly drinking unadjusted OR**	***p***	**Weekly drinking adjusted OR**	***p***

**N of off-sales within 1200 m**								
0–10	1.00		1.00		1.00		1.00	
11–20	1.25 (0.85–1.84)	0.249	1.13 (0.76–1.68)	0.534	1.20 (0.80–1.80)	0.386	1.07 (0.71–1.63)	0.740
21–30	1.40 (0.96–2.05)	0.084	1.36 (0.92–2.01)	0.121	1.28 (0.85–1.93)	0.232	1.23 (0.81–1.87)	0.335
31+	1.60 (1.09–2.36)	0.016	1.49 (1.00–2.21)	0.046	1.54 (1.02–2.32)	0.041	1.43 (0.94–2.19)	0.095
**Social background**								
**Family (2-parent)**	1.00		1.00		1.00		1.00	
1-Parent	0.49 (0.32–0.77)	0.002	0.52 (0.33–0.82)	0.005	0.50 (0.32–0.79)	0.003	0.54 (0.34–0.85)	0.007
Reconstituted/Other	0.54 (0.40–0.74)	≤0.001	0.45 (0.32–0.64)	≤0.001	0.55 (0.40–0.76)	≤0.001	0.46 (0.32–0.66)	≤0.001
**Social class (non-manual)**	1.00				1.00		1.00	
Manual	0.97 (0.65–1.43)	0.861	1.35 (0.87–2.07)	0.177	0.91 (0.61–1.36)	0.643	0.69 (0.51–0.94)	0.020
Missing	0.73 (0.55–0.99)	0.042	0.72 (0.53–0.98)	0.036	0.70 (0.52–0.95)	0.020	1.28 (0.83–1.98)	0.265
**Gender (male)**	1.40 (1.07–1.83)	0.015	1.30 (0.99–1.72)	0.063	1.42 (1.08–1.86)	0.011	1.32 (0.99–1.74)	0.055

## 5 Discussion

We found mixed support linking accessibility of alcohol outlets and alcohol use among our Scottish adolescent population. Irrespective of gender, clear and significant associations were found between some measures of accessibility such as proximity to specific types of outlets (off-sales) and high alcohol consumption among adolescents. In contrast, proximity and outlet density of on-premise outlets (i.e. local pubs or clubs) was unrelated to adolescent alcohol use, nor was off-sales outlet density measured at the very immediate neighbourhood level (data zone). Our results challenge the relatively clear conclusion drawn by The (American) Task Force on Community Preventive Services (2009) which considered the association between alcohol density and alcohol use robust, but is compatible with a recent systematic review which, after evaluating recent evidence, is more cautious in its conclusions (Bryden et al., 2011). The review acknowledged that higher outlet density may be associated with higher alcohol use, particularly among adolescents, but that the current evidence base to support this claim was weak.

The lack of association between the availability of on-premise drinking venues and adolescent alcohol use may be due to the greater price per unit of alcohol and stringent enforcement of age-restrictions associated with on-premise outlets. As this is the first study in Scotland to examine the relationship between proximity to off-sales outlet and regular alcohol use by underage drinkers, we briefly discuss its relevance for theory and public policy, before considering its limitations.

### 5.1 Theory

Our results offer partial support for Campbell's et al. model (Campbell et al., 2009) of alcohol availability and alcohol use (Fig. 1), confirming the importance of one such pathway: density of off-premises outlets—leads to decreased distance to local outlets—leads to increased access to alcohol—leads to higher alcohol consumption, but not other pathways such as that between on-premises outlet density and increased consumption. While conceptually useful, the model omits many contextual influences, not least how the importance of each pathway alters throughout the life course. The illustrated path model also obfuscates certain aspects of outlet density that are highly relevant. For example, ‘distance to alcohol outlet’ does not capture the effects of outlet clustering and a high outlet density does not necessarily mean a decreased distance to outlets.

### 5.2 Public policy

From a public policy perspective, our findings suggest restricting the number of off-sales outlets and increasing the distance to outlets might reduce the level of alcohol consumption among adolescents, e.g. by limiting the number of outlets in high residential areas or concentrating outlets in city centres. Conversely, while proximity to off-licensed premises was related (in a non-linear manner) to greater alcohol use, *density* of off-sales outlets within the immediate neighbourhood (data zone) showed no association with drinking patterns. The number of off-sales within 1200 m was marginally associated with weekly alcohol use. This measure combines both proximity and local density and can arguably be considered a crude measure of local outlet clustering. These discrepant findings may be potentially explained by distinguishing among three types of contextual effects; *proximity* (how easily one can access alcohol); *amenity* (how outlets influence the quality and characteristics of neighbourhood) and *outlet cluster* (locations with multiple outlets in very close proximity).

In relation to the first two effects, Livingston (Livingston et al., 2007) suggests there is a non-linear relationship between outlets and problematic alcohol use, with amenity effects showing an exponential (*J*-curve) relationship and proximity effects showing the inverse (inverse *J*-curve). We found evidence for a proximity, but not amenity, effect. If we anticipate nonlinear effects, detecting the critical threshold distance appropriate for lifestage and context is an important consideration. We found the association with alcohol consumption significant only at very close distances, while others using predominantly adult samples, found effects at larger distances than those used in the present study (Halonen et al., 2012, Kypri et al., 2008, Truong and Sturm, 2007)

Another potential (local density) effect we have not directly measured, but which is compatible with our proximity results, is that of outlet clustering. Living very close to an alcohol outlet, or many nearby outlets could be a proxy indicator for residing near such a cluster. Alternatively, this could indicate differences in the pattern of outlet clustering within different ‘neighbourhood types’, e.g. a divergent pattern of outlet clustering in equally deprived inner city and peripheral neighbourhoods (Ellaway et al., 2010). These clusters may be local ‘hot-spots’ for alcohol-related trouble, particularly that involving underage drinkers and may provide adolescents easy access to alcohol either purchased directly from outlets or from peers who congregate in that area (Campbell et al., 2009). Outlet clusters may provide adolescents with a greater choice of outlets to which multiple illegal purchase attempts can be made within a short time. This raises a further policy concern; despite good intentions policies to reduce alcohol outlet density could have serious unintended consequences (Campbell et al., 2009). For example, drastically reducing the number of alcohol outlets could encourage large groups of adolescents to gather near the few remaining outlets, unintentionally creating new ‘hotspots’.

This complex relationship between local outlets and young people’s purchasing and drinking behaviour is illustrated in a qualitative study of street drinking Glaswegian youth (Galloway et al., 2007). The study found many young people drink on public transport while travelling towards the city centre where they can purchase additional alcohol or engage in street drinking. Paradoxically some young people preferred public drinking in the city centre because of the perceived safety and heavy CCTV surveillance. Consequently restricting the number of local outlets may have only a limited effect on young people’s drinking behaviour. That said, there is evidence that underage drinkers target smaller off-licenses with less rigorous selling practices outwith their own local area (but not within the city centre) to avoid stigma and increase their chances of purchase. This was primarily because supermarkets and larger off-licences stores were perceived as enforcing stricter selling practices. Given this complex purchasing and consumption pattern, we do not expect a simple direct link between outlet proximity or density measures and alcohol consumption. Thus a successful outlet-based intervention would likely have to be both integrated and citywide to have maximum effect, yet may still have unintended social consequences, i.e. restricting the number of local shops with alcohol licences may well reduce young people's access to alcohol, but result in many local shops closing, further reducing already limited facilities in deprived areas.

### 5.3 Limitations

Despite a number of strengths, this study has several methodological shortcomings. While participants were subsampled from a larger more representative study, heterogeneous in social background and location, it is not a random geographical sample and we are appropriately cautious in generalising results. Our indicator of social class is derived from pupils' reports of parental occupation and not from parents directly, nevertheless this method has provided valid data and social class derived from pupils as young as age 11 is highly correlated with that derived from parental self-report (West et al., 2001). Our other measures are limited, not least because we do not track adolescents ‘actual routes’ or actual exposure to outlets. A recent small scale American study compared adolescents exposure to alcohol outlets (as measured by distance to outlets from residence) with exposure measured by tracking the ‘actual’ route taken in a 24 h period of activity, finding scant overlap between the two, nor with exposure calculated using their administratively defined (census tract) neighbourhood (Basta et al., 2010). Further, our measures do not fully capture adolescents' opportunity to access alcohol, e.g. adolescents may have access to alcohol within the family home, either with or without parental consent or they may obtain alcohol from city centre outlets.

Our study, like most others in this area, is cross-sectional and accordingly it cannot imply causation between proximity/density and alcohol use. A recent longitudinal study of over 1000 Californian adolescents following their individual ‘access to alcohol’ trajectories over 3 years challenges such simple causal assumptions (Chen et al., 2009). It found, while outlet density at the zip code level predicted the initial level of access, density had little impact on the pattern of access over the subsequent 3 years and the results suggest that greater outlet density leads to *slower* growth in adolescence access to alcohol. Finally, we assessed the impact of outlet availability on only a single alcohol outcome, i.e. frequency of drinking. Pollack et al. (2005) suggest that not all alcohol outcomes are associated with every measure of availability, for example while alcohol consumption might be linked to proximity, alcohol-related problems may be predominantly associated with density of outlets . Thus different alcohol outcomes, such as binge drinking, quantity or type of alcohol purchased are likely to be linked with different outlet characteristics. This may in part explain the mixed findings within the literature and is an area worth exploring further.

## 6 Conclusion

Campbell et al. (2009) outline the pathways between seven characteristics that potentially influence the impact of alcohol outlets on health: outlet type and number of outlets; outlet size; clustering of outlets; proximity to places of concern (i.e. schools); outlets associated with illegal activity (i.e. drug use); the size of neighbourhood; neighbourhood characteristics (i.e. demographics). Here we explored the influence of the first and last of these factors finding both to be important predictors of adolescents alcohol consumption and that fine grained individual-level measures of exposure may be necessary to detect such effects.

## Disclosure of interests

All authors are supported financially by the Medical Research Council of Great Britain as part of the Neighbourhoods and Health programme at the MRC/CSO Social and Public Health Sciences Unit. All authors declare no competing interests. All authors state they have no financial disclosures
